# A sensitive and compact optical detector based on digital lock-in amplification

**DOI:** 10.1016/j.ohx.2021.e00228

**Published:** 2021-09-02

**Authors:** Andrew J. Harvie, Surendra K. Yadav, John C. de Mello

**Affiliations:** Department of Chemistry, NTNU, Trondheim, Norway

**Keywords:** Bioassays, Fluorescence, 3D printing, Open hardware, Digital signal processing, Lock-in

## Abstract

We report a sensitive, fixed-wavelength, lock-in-based optical detector built from a light-emitting diode, two colour filters, a photodetector, a small number of discrete analogue components, and a low-cost microcontroller development board. We describe the construction, operating principle, use and performance of the optical detector, which may be used for both absorption and fluorescence measurements in either a 10-mm pathlength cuvette or a low-volume (<100 μl) flow-cell. For illustrative purposes the detector is applied here to a cholesterol assay based on the enzyme-mediated conversion of (non-emissive) Amplex Red into the fluorescent dye resorufin, providing a detection limit of ~200 nM – some four orders of magnitude lower than the typical concentration of cholesterol in human serum. (The resorufin molecule itself is detectable down to concentrations of ~20 nM). The system may be readily adapted to other biomolecules through a simple change of enzyme.

## Graphical abstract

**Figure:**
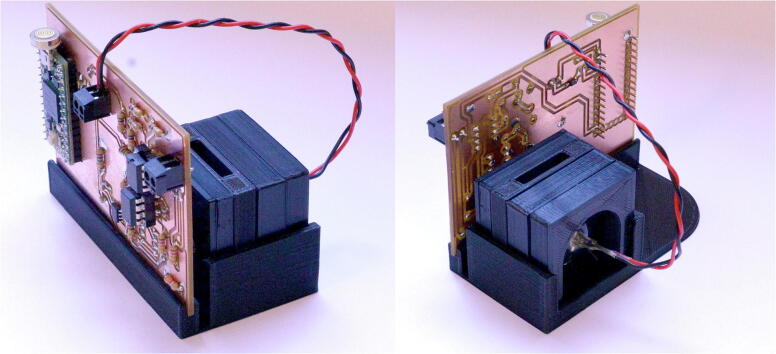

**Specifications table:** 

Hardware name	Lock-In Luminometer
Subject area	Chemistry and Biochemistry
Hardware type	Measuring physical properties and in-lab sensors
Closest commercial analogue	Luminometer
Open Source Licence	MIT
Cost of Hardware	< £135
Source File Repository	https://doi.org/10.5281/zenodo.5106522

## Hardware in context

The detection and quantitation of weak, slowly varying fluorescence signals from trace concentrations of chromophores is an essential task in many fields of science. Important examples include: biochemistry, where low-concentration biomolecules are quantified using fluorogenic assays; [1] synthetic chemistry, where reaction kinetics are often determined by monitoring changes in fluorescence intensity due to consumed or generated fluorophores; [2] and environmental science, where fluorescence is used to detect and quantify pollutants, either directly or indirectly via their interaction with probe dyes [3], [4], [5].

In common with other optical signals, fluorescence is commonly monitored using a photodiode coupled to a sensitive current meter, with the current generated by the photodiode typically being proportional to the intensity of the incident light. At low fluorophore concentrations, the weak fluorescence signal is frequently masked by noise and/or strong background signals, preventing reliable detection and measurement. In such circumstances, the fluorescence signal may often be extracted by modulating the light-source (and therefore the fluorescence signal) at a fixed frequency $f_{mod}$, and using a signal processing technique known as “lock-in” detection [6] to eliminate unwanted interferences at other frequencies. The lock-in technique is simple to implement and, as shown here, can give good performance even with inexpensive hardware, making it an attractive choice for low-cost, high sensitivity fluorescence detection.

In this manuscript we describe a sensitive, fixed-wavelength, lock-in-based fluorescence detector built from a light-emitting diode (LED), two colour filters, a photodetector, a small number of discrete analogue components, and a low-cost microcontroller development board. We describe the construction, operating principle, use and performance of the fluorescence detector, which allows measurements to be carried out in a 10-mm pathlength cuvette or a low volume (<100 μl) flow-cell. For illustrative purposes the detector is applied here to a cholesterol assay based on the enzyme-mediated conversion of (non-emissive) Amplex Red into the fluorescent dye resorufin [1]. The system may be readily adapted to other biomolecules through a simple change of enzyme.

## Hardware description

**Fig. 1:**
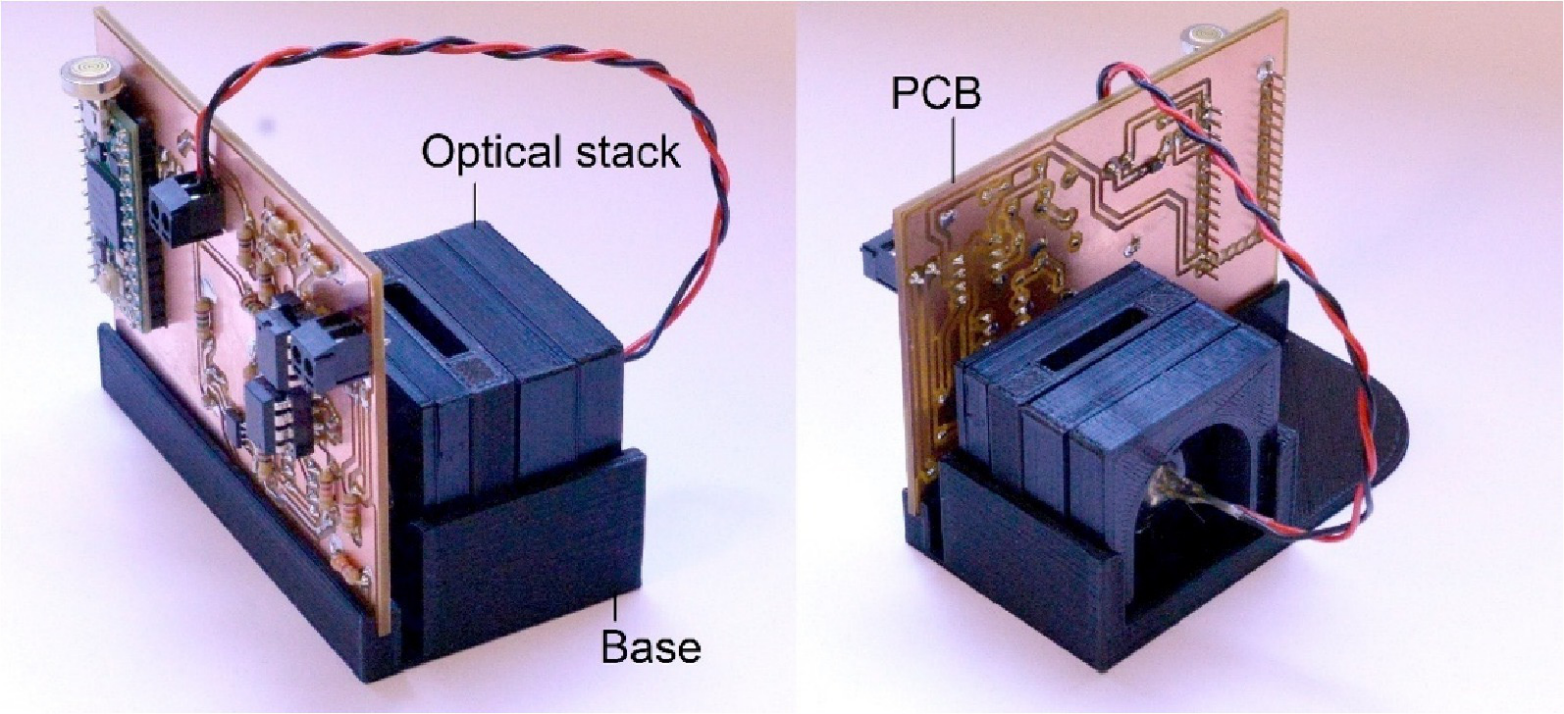
Rear- and front-facing photographs of assembled fluorimeter. The device consists of three main parts: a PCB for the detection electronics, a 3D-printed housing for the optical components, and a 3D-printed base-plate for mechanical support. An LED inside the housing is used as a light-source, while an amplified photodiode mounted on the rear side of the PCB is used as a detector.

The hardware (see photographs in Fig. 1) comprises three main parts: (i) a printed circuit board (PCB), onto which all electronic components except the LED light-source are mounted; (ii) a 3D-printed housing for the LED, optical filters and sample; and (iii) a 3D-printed base-plate that holds the optics and PCB together. The PCB contains power management circuitry, an LED driver, an amplified photodiode to detect the emitted light, analogue circuitry for signal conditioning, and a microcontroller development board for carrying out analogue to digital conversion and lock-in detection.

### Optical detection

**Fig. 2:**
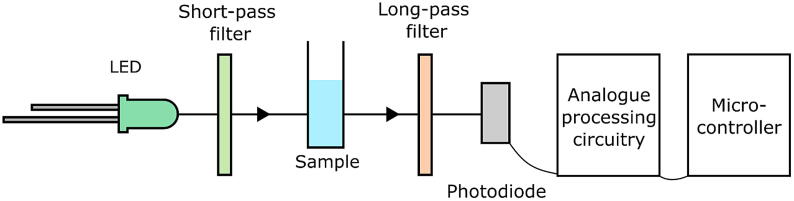
Simplified schematic of optical setup. Light from an LED passes through a short-pass filter and strikes the sample, which is held in a 10-mm pathlength cuvette or a simple flow cell. Emitted light from the sample plus transmitted light from the LED strikes a long-pass filter that allows only the emitted light to pass and reach the amplified photodiode. The signal from the photodiode is pre-processed using analogue circuitry and then passed to a microcontroller development board for analogue to digital conversion and digital signal processing.

The optical configuration is shown schematically in Fig. 2. Excitation light from the LED passes through a short-pass filter, which absorbs long-wavelength photons that overlap spectrally with the fluorophore emission. The filtered light from the LED passes through a detection zone containing the sample under test, where it is partially absorbed and reradiated by the fluorophores. The emergent light from the sample – a mixture of unabsorbed light from the LED and longer-wavelength light from the emitting fluorophores – passes through a long-pass filter, which heavily attenuates the excitation light but allows the emitted light to pass and strike the photodetector. The sample is introduced into the detection zone using a 10-mm cuvette or a low-volume (<100 μl) flow cell. Interchangeable 3D-printed holders allow for easy swapping between cuvette- and flow-based detection. (The greater sample volume of the cuvette typically provides slightly better sensitivity but requires sample volumes of at least 2 ml compared to ∼100 μl for the flow cell). The holders may be readily adapted to other sample formats.

To reduce size, minimise the parts count, and simplify assembly, we do not use discrete focussing optics to guide the exciting or emitted light, although the chosen LED has a focussing lens integrated within its housing. If required, additional lenses could be straightforwardly incorporated into the housing for better light management and enhanced sensitivity. By replacing the short- and long-pass filters with band-pass filters matched to the emission peak of the LED, the system may be straightforwardly adapted to absorption-based measurements. (The ability to switch between fluorescence- and absorption-based measurements is the reason why we have chosen to arrange the light-source and detector in a face-to-face configuration rather than at 90° to one another, although it would be simple to adapt the system to orthogonal fluorescence detection if required).

**Fig. 3:**
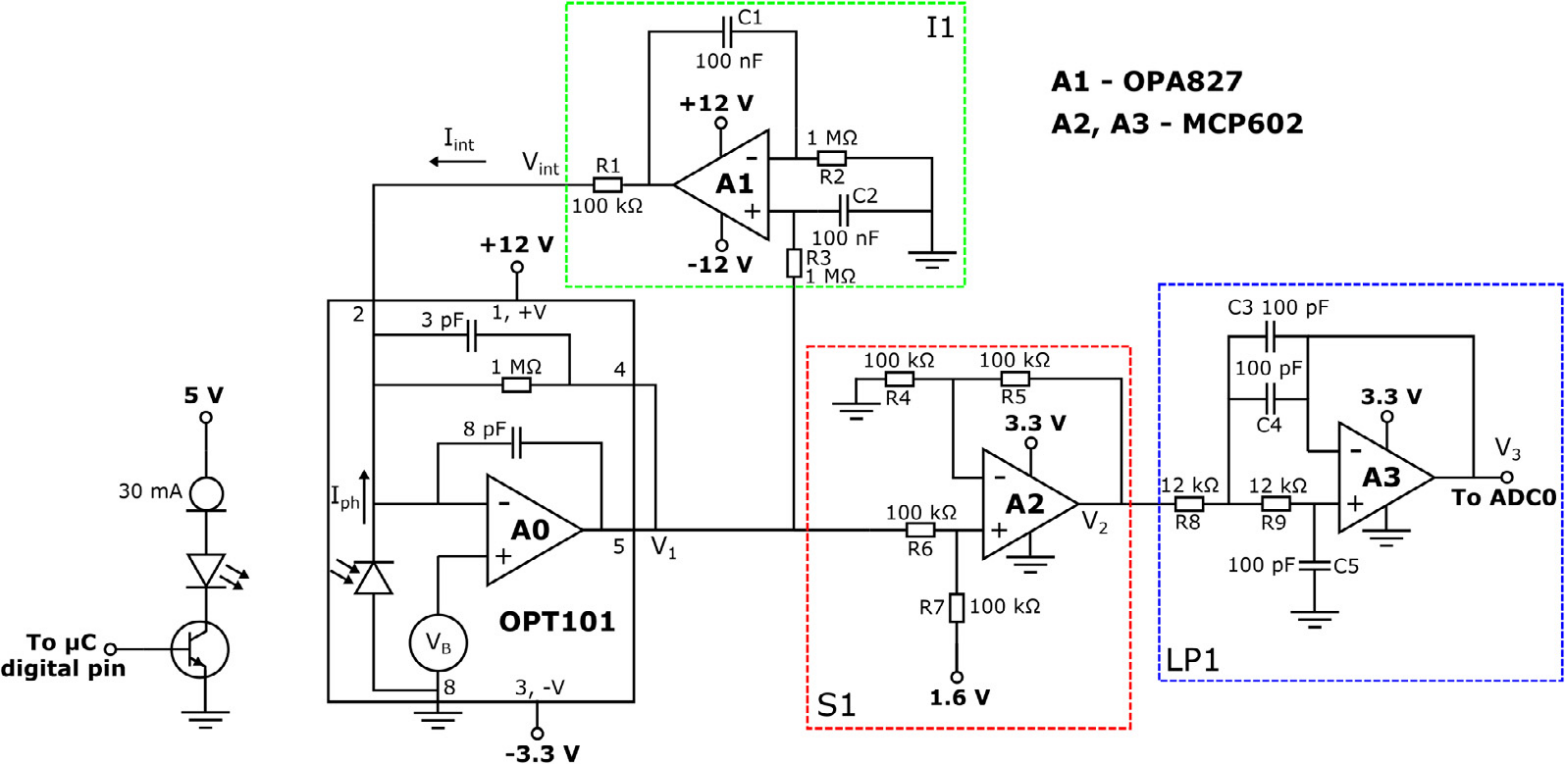
Schematics of LED driver and analogue conditioning circuit. The LED is connected in series with a 30-mA constant current diode and an NPN transistor; the base terminal of the transistor is connected to a digital output pin of the microcontroller, which switches the current flow on and off at a frequency of ~410 Hz and a duty cycle of 50%. The optical signal is detected using an amplified photodiode (OPT101). The DC output $V_{1}$ from the amplified photodiode is passed through a non-inverting integrator (I1, green box) that generates a positive output current $I_{int}$ at the summing terminal of A0 (Pin 2 of the OPT101), cancelling the negative DC photocurrent due to ambient light and hence reducing the DC component of $V_{1}$ to zero. $V_{1}$ is passed to a non-integrating amplifier that adds a constant DC offset of 1.6 V, yielding an output voltage $V_{2}$ that spans the range 0 to 3.2 V (S1, red box). Finally, $V_{2}$ is passed to an active second-order low-pass filter (LP1, blue box) that suppresses noise and high-frequency components above 94 kHz. (For interpretation of the references to colour in this figure legend, the reader is referred to the web version of this article.)

For the purposes of cholesterol detection, the LED is chosen to have an emission peak at 525 nm, which coincides with the absorption band of the resorufin dye used in the enzymatic assay. The Full Width Half Maximum (FWHM) of the LED’s emission spectrum is approximately 25 nm, but a long-wavelength tail extends beyond 570 nm (Fig. S1a). The emission tail is attenuated by the short-pass filter, which has a cut-off at 550 nm (Fig. S1b). The emission band of resorufin lies predominantly in the range 550 – 650 nm, with a weak tail that extends to longer wavelengths (Fig. S1a). The long-pass filter has a cut-on at 570 nm (Fig. S1b) and therefore blocks most of the short-pass-filtered excitation light, while efficiently passing the emitted light. The LED is modulated at ~410 Hz, using a 30–mA constant-current driver circuit (see Fig. 3) that is toggled on and off with a 50% duty cycle using a digital output pin of the microcontroller. For the 30-mA square-wave modulation used here, the time-averaged output power of the LED is just over 1 mW. The amplified silicon photodiode (OPT101) has a broadband optical response from 400 to 1000 nm, which fully covers the emission band of resorufin (Fig. S1c). The set-up may be readily adapted to assays based on other dye molecules by swapping the LED and optical filters as needed.

### Analogue signal processing

The amplified photodiode (OPT101) is used in dual-supply mode with a transimpedance gain of −1 V/μA. The output $V_{1}$ of the amplified photodiode feeds into a three-stage signal-conditioning circuit that prepares the signal for read-out by the microcontroller’s built-in analogue-to-digital converter ADC0 (which accepts input voltages in the range 0 to 3.3 V). The first stage of the conditioning circuit is a feedback-based compensation stage that removes any DC offset in $V_{1}$ due to ambient light or non-ideality of A0, allowing the OPT101 to operate under conditions of high ambient illumination without saturating ADC0. (Note, the compensation circuit treats all frequencies below 16 Hz as DC and hence the modulation frequency must be substantially higher than 16 Hz for a reliable measurement. For the present set-up, we use a fixed modulation frequency of ~410 Hz). The second stage of the conditioning circuit is a summing amplifier (S1) that converts an input signal in the range −1.6 V to + 1.6 V into an output signal in the range 0 V to 3.2 V, allowing it to be sampled by ADC0. The third stage is a unity-gain low-pass filter with a cut-off frequency of 94 kHz, slightly less than half the 200-kHz sample rate used by ADC0. The low-pass filter attenuates noise and interferences above the 100-kHz Nyquist frequency of ADC0, preventing sampling artefacts due to aliasing. Further details of the conditioning circuit are provided in the Supporting Information.

### Digital signal processing

**Fig. 4:**
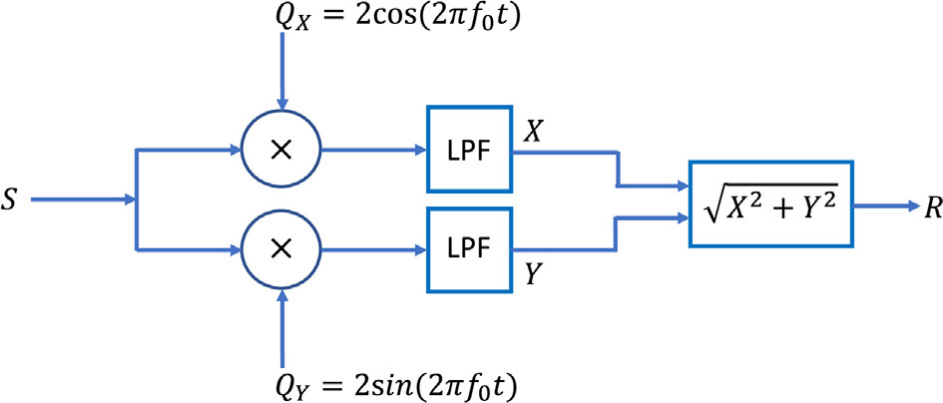
Block diagram illustrating lock-in procedure. An input signal S of frequency $f_{0}$ is passed into two separate channels. In the first channel S is multiplied by a reference signal of the form $2\cos\left(2\pi f_{0}t\right)$ and passed through a low-pass filter (LPF), yielding an intermediate output X, while in the second channel it is multiplied by a reference signal of the form $2\sin\left(2\pi f_{0}t\right)$ and passed through a second low-pass filter, yielding an intermediate output Y. X and Y are vectorially combined to yield a final output $R=\sqrt{X^{2}+Y^{2}}$ that is proportional to the amplitude of input signal S.

Analogue to digital conversion and digital signal processing is carried out on a Teensy 4.0 microcontroller development board (PJRC). The Teensy 4.0 has a fast 600-MHz ARM processor with on-board Floating Point Unit (FPU), allowing it to efficiently carry out the calculations needed to implement digital lock-in detection. In brief, a sampled periodic signal S of frequency $f_{0}$ is multiplied by sinusoidal and cosinusoidal reference signals of matching frequency $f_{0}$, and the resulting output signals are passed through separate digital low-pass filters to remove non-DC components (see the block diagram in Fig. 4). Following low-pass filtering we obtain two scalar outputs X and Y derived from the two reference signals. X and Y are vectorially combined to obtain an output signal $R=\surd (X^{2}+Y^{2})$ that is directly proportional to the amplitude of S. (Formally, R corresponds to the amplitude of the first harmonic of S). The process repeats every time a new sample is acquired, so the lock-in outputs X, Y and R are updated at the sample rate of the ADC. R is affected only by input signals that are very close in frequency to $f_{0}$ so the target signal S may be reliably measured even if it is corrupted by significant noise and/or interferences at other frequencies. A detailed description of the lock-in method is provided in the Supporting Information.

Computationally, the lock-in procedure is carried out as follows. An incoming analogue signal of frequency $f_{0}$ (together with noise and interfering signals at other frequencies) is sampled at discrete times $t\left(1\right),t\left(2\right),t\left(3\right)\cdots$ where the time interval Δt between successive measurements is fixed at 5 μs. Timing errors are minimised by choosing $f_{0}$ to be an exact integer fraction of the sample rate $f_{s}$, i.e. $f_{0}=f_{s}/m=1/\left(m\Delta t\right)$ where m is an integer. Here, we choose a sample rate of 200 kHz and an m-value of 488, which corresponds to $f_{0}$ = 409.84 Hz. The sample values $S\left(1\right),S\left(2\right),S\left(3\right)\cdots$ are obtained from the ADC as 12-bit integers and stored as 64-bit doubles. All calculations are carried out at 64-bit precision using the microcontroller’s onboard FPU. Immediately after each sample S(n) has been acquired, the two reference signals $Q_{x}\left(n\right)$ and $Q_{y}\left(n\right)$ are updated as follows:

$$Q_{x}\left(n\right)=\cos\left(2\pi f_{0}n\Delta t\right)=cos\left(2\pi n/m\right)$$

and

$$Q_{y}\left(n\right)=\sin\left(2\pi f_{0}n\Delta t\right)=sin\left(2\pi n/m\right),$$

where the sample number n = 1, 2, 3… The LED driver is toggled on or off every m/2 steps, i.e. whenever $Q_{y}\left(n\right)=0$, resulting in a fluorescence signal that is phase-locked to the reference signals and of identical frequency. (Note, the exact phase of the fluorescence signal relative to the reference signals depends on the turn-on time of the LED driver and varies according to the modulation frequency. The use of two reference signals that differ in phase by 90° corrects for the effects of the slow LED turn-on).

Multiplying $Q_{x}\left(n\right)$ and $Q_{y}\left(n\right)$ by S(n) we obtain two (unfiltered) intermediate outputs $X_{0}\left(n\right)$ and $Y_{0}\left(n\right)$:

$$X_{0}\left(n\right)=Q_{x}\left(n\right)S\left(n\right)$$

and

$$Y_{0}\left(n\right)=Q_{y}\left(n\right)S\left(n\right).$$

The low-pass filtering step is carried out by exponentially averaging $X_{0}\left(n\right)$ and $Y_{0}\left(n\right)$, [7] which yields two intermediate outputs $X_{1}\left(n\right)$ and $Y_{1}\left(n\right)$:

$$X_{1}\left(n\right)=X_{1}\left(n-1\right)+\alpha \left[X_{0}\left(n\right)-X_{1}\left(n-1\right)\right]$$

and

$$Y_{1}\left(n\right)=Y_{1}\left(n-1\right)+\alpha \left[Y_{0}\left(n\right)-Y_{1}\left(n-1\right)\right],$$

where $X_{1}\left(n-1\right)$ and $Y_{1}\left(n-1\right)$ are the previous values of $X_{1}$ and $Y_{1}$ and α is a weighting coefficient that determines the cut-off frequency of the exponential filter. ($X_{1}$ and $Y_{1}$ are arbitrarily set to zero prior to the first sample). α is set to a constant value of 7.85 × 10^-6^, which corresponds to a cut-off frequency $f_{c}$ of 0.25 Hz and a time-constant τ of 0.63 s.[7] The exponential filtering stage is repeated using the same α value to provide better attenuation of non-DC interferences and hence a ‘cleaner’ solution:

$$X_{2}\left(n\right)=X_{2}\left(n-1\right)+\alpha \left[X_{1}\left(n\right)-X_{2}\left(n-1\right)\right]$$

and

$$Y_{2}\left(n\right)=Y_{2}\left(n-1\right)+\alpha \left[Y_{1}\left(n\right)-Y_{2}\left(n-1\right)\right]$$

Finally, $X_{2}\left(n\right)$ and $Y_{2}\left(n\right)$ are combined vectorially to obtain a single scalar output $R_{2}\left(n\right)$:

$$R_{2}\left(n\right)=\sqrt{X_{2}\left(n\right)X_{2}\left(n\right)+Y_{2}\left(n\right)Y_{2}\left(n\right)}$$

$R_{2}\left(n\right)$ is proportional to the amplitude of the first harmonic of the target signal S (see Supporting Information) which, in practical terms, means it is proportional to the average fluorescence intensity of the sample under test (plus a background contribution from residual LED light that leaks through the long-pass filter). Interferences from spurious signals at other frequencies are greatly attenuated by the lock-in algorithm (see Supporting Information).

The microcontroller sends out a new $R_{2}$ value over its universal serial bus (USB) interface every 0.1 s for remote analysis on a PC or other hardware. For a reliable measurement, it is advisable to wait 10 time constants after switching on the sensor, loading a new sample, or changing a parameter for the lock-in output to stabilise. Hence, for the 0.63 s time constant used here, a wait time of 6–7 s is recommended. A simple PC-based software front-end is provided for reading, plotting and saving data (see operating instructions).

### Power management

The PCB has two sets of bipolar power rails at ± 3.3 V and ± 12 V. The ± 3.3 V rails are powered by the Teensy’s built-in 3.3 V supply (which in turn is powered via the USB connection), using a TC7660 inverting charge pump IC on the PCB to feed the −3.3 V rail. The ±12 V rails are derived from an external 24 V unipolar supply, using a resistive divider with its midpoint connected to the ground of the 3.3 V unipolar supply. The Teensy microcontroller and the (single-supply) MCP602 dual op-amp IC used in S1 and LP1 are operated at +3.3 V. The OP827 op-amp in the DC compensation circuit is operated at ±12 V, and the OPT101 amplified photodiode is powered using a combination of the +12 V and –3.3 V rails to allow for bipolar operation around 0 V.

### Main features of optical detector at-a-glance

• Easy measurement of fluorescence in a flow-cell or 10-mm-pathlength cuvette
• Excellent rejection of ambient light, noise and other interferences
• High sensitivity and linearity over a wide range of intensities
• Applicable to a wide range of fluorogenic biochemical assays
• Modular design can be customised to accept a wide range of samples, LEDs and filters

## Design files

Table 1, Table 2 contain details of the design files and software code. A complete bill of materials may be found in Table 3.

**Table 1:** Summary of design files.

Design file name	File type	Open source licence	Location of the file
Base.stl	CAD file – 3D print	MIT Licence	https://doi.org/10.5281/zenodo.5106522
EmissionPlate.stl	CAD file – 3D print	MIT Licence	https://doi.org/10.5281/zenodo.5106522
ExcitationPlate.stl	CAD file – 3D print	MIT Licence	https://doi.org/10.5281/zenodo.5106522
FlowCellHalf1.stl	CAD file – 3D print	MIT Licence	https://doi.org/10.5281/zenodo.5106522
FlowCellHalf2.stl	CAD file – 3D print	MIT Licence	https://doi.org/10.5281/zenodo.5106522
FlowCellHolder.stl	CAD file – 3D print	MIT Licence	https://doi.org/10.5281/zenodo.5106522
PDPlate.stl	CAD file – 3D print	MIT Licence	https://doi.org/10.5281/zenodo.5106522
CuvetteHolder.stl	CAD file – 3D print	MIT Licence	https://doi.org/10.5281/zenodo.5106522
RetainingClip.stl	CAD file – 3D print	MIT Licence	https://doi.org/10.5281/zenodo.5106522
CuvetteCap.stl	CAD file – 3D print	MIT Licence	https://doi.org/10.5281/zenodo.5106522
PCB.brd	CAD file - PCB	MIT Licence	https://doi.org/10.5281/zenodo.5106522
PCBGerbers.zip	CAM files - PCB	MIT Licence	https://doi.org/10.5281/zenodo.5106522
FluorescenceSensor.ino	Microcontroller firmware	MIT Licence	https://doi.org/10.5281/zenodo.5106522
SensorInterface.py	Software frontend	MIT Licence	https://doi.org/10.5281/zenodo.5106522
widget.ui	Software frontend component	MIT Licence	https://doi.org/10.5281/zenodo.5106522
communicator.py	Software frontend component	MIT Licence	https://doi.org/10.5281/zenodo.5106522

**Table 2:** Design file description.

Base.stl	STL model file for the 3D-printed base.
EmissionPlate.stl	STL model files for the 3D-printed components in the optical stack (see Build Instructions for further details).
ExcitationPlate.stl	
FlowCellHolder.stl	
PDPlate.stl	
RetainingClip.stl	
FlowCellHalf1.stl	STL model files for the 3D-printed parts used to construct the liquid flow cell.
FlowCellHalf2.stl	
CuvetteHolder.stl	STL model file for the 3D-printed cuvette holder.
CuvetteCap.stl	STL model file for an optional cap to protect the cuvette from external light.
PCB.brd	BRD (Autodesk Eagle) format design files for the printed circuit board.
PCBGerbers.zip	Collated Gerber and drill files for PCB manufacture. An external manufacturer will likely require these files, packaged within the .zip archive.
FluorescenceSensor.ino	Arduino source code for the microcontroller development board. Arduino and Teensyduino must be installed on the control PC.
SensorInterface.py	Python script for the software frontend. Requires installation of Python 3.x or higher, the PyQt5, pyqtgraph and PySerial libraries, plus widget.ui and communicator.py to run.

**Table 3:** Bill of materials.

Item	Unit cost	Total cost	Source	Material type
Teensy 4.0 × 1	£18.50	£18.50	https://coolcomponents.co.uk/products/teensy-4–0-usb-development-board	Other
PCB × 1	∼£2	∼£2	Self-made	Composite
3D-printed parts:		∼£2	Self-made	Polymer
Base × 1				
Retaining clip × 1				
Excitation plate × 1				
Emission plate × 1				
Photodiode plate × 1				
Flow cell mount × 1				
Flow cell halves × 2				
8-pin DIP socket × 1	£0.62	£0.62	https://uk.rs-online.com/web/p/dil-sockets/0813115/	Other
TC7660EPA charge pump IC × 1	£0.80	£0.80	https://uk.rs-online.com/web/p/voltage-regulators/7747876/	Semiconductor components
MCP602-I/P op-amp IC × 1	£0.48	£0.48	https://uk.rs-online.com/web/p/op-amps/3792572/	Semiconductor components
OPA827AID op-amp IC × 1	£12	£12	https://uk.rs-online.com/web/p/op-amps/7093267/	Semiconductor components
OPT101 amplified photodiode × 1	£6	£6	https://uk.rs-online.com/web/p/photodetector-amplifiers/1977465	Semiconductor components
0805 10 μF capacitor × 2	£0.14	£0.28	https://uk.rs-online.com/web/p/mlccs-multilayer-ceramic-capacitors/9155225/	Ceramic
0805 100 nF capacitor × 2	£0.05	£0.10	https://uk.rs-online.com/web/p/mlccs-multilayer-ceramic-capacitors/4646688/	Ceramic
0805 100 pF capacitor × 3	£0.10	£0.30	https://uk.rs-online.com/web/p/mlccs-multilayer-ceramic-capacitors/0391000/	Ceramic
Resistors (through hole):		£1	Generic	Any type
3.3 kΩ × 2				
12 kΩ × 2				
10 kΩ × 2				
100 kΩ × 5				
1 MΩ × 2				
NSI45030AT1G 30 mA LED driver × 1	£0.15	£0.15	https://uk.rs-online.com/web/p/display-drivers/7377990P/	Semiconductor components
BC547B NPN transistor × 1	£0.14	£0.14	https://uk.rs-online.com/web/p/bipolar-transistors/7619828P/	Semiconductor components
FGL570 570 nm coloured long-pass glass filter × 1	£20.69	£20.69	https://www.thorlabs.com/thorproduct.cfm?partnumber = FGL570	Glass optic
FES0550 550 nm short-pass filter × 1	£60.47	£60.47	https://www.thorlabs.com/thorproduct.cfm?partnumber = FES0550	Glass optic
LED525L green LED × 1	£9.41	£9.41	https://www.thorlabs.com/thorproduct.cfm?partnumber = LED525L	Semiconductor components
3.5 mm PCB terminal block × 2	£0.36	£0.72	https://uk.rs-online.com/web/p/pcb-terminal-blocks/8971332/?sra = pstk	Other
		**£135.66**		

**Comment on costs –** We note that the system was assembled from parts that we already had to hand in our laboratory, and there is substantial scope for reducing the overall bill of materials through judicious component replacements. The Teensy 4.0, OPA827, LED and colour filters together account for 88% of the bill of materials. At the time of writing, all items except the Teensy 4.0 microcontroller could be replaced by substantially cheaper alternatives without requiring any significant design changes. The £12 OPA827 op-amp used in the DC compensation circuit could be replaced by a variety of lower cost DC servo op-amps such as the Texas Instruments TL072CP, which costs approximately £1. The LED525L green LED could be replaced by a wide range of high brightness generic LEDs with similar optical output power and spectral characteristics, which are widely available for £1 or less. The long- and short-pass filters could be replaced by suitably chosen plastic colour filters at a cost of a few pounds. (Coloured ‘gels’ for stage lighting are a good source of low-cost colour filters, while home-made dye-sensitised titania filters are an attractive option for high-sensitivity applications where filter fluorescence must be minimised [8], [9]). By making these simple component substitutions, we consider it feasible to reduce the total bill of materials to around £45 without reducing the overall performance of the system. (Indeed switching to a more optimal pairing of short- and long-pass filters can be expected to improve performance, see Fig. S1b).

### Build instructions

**Fig. 5:**
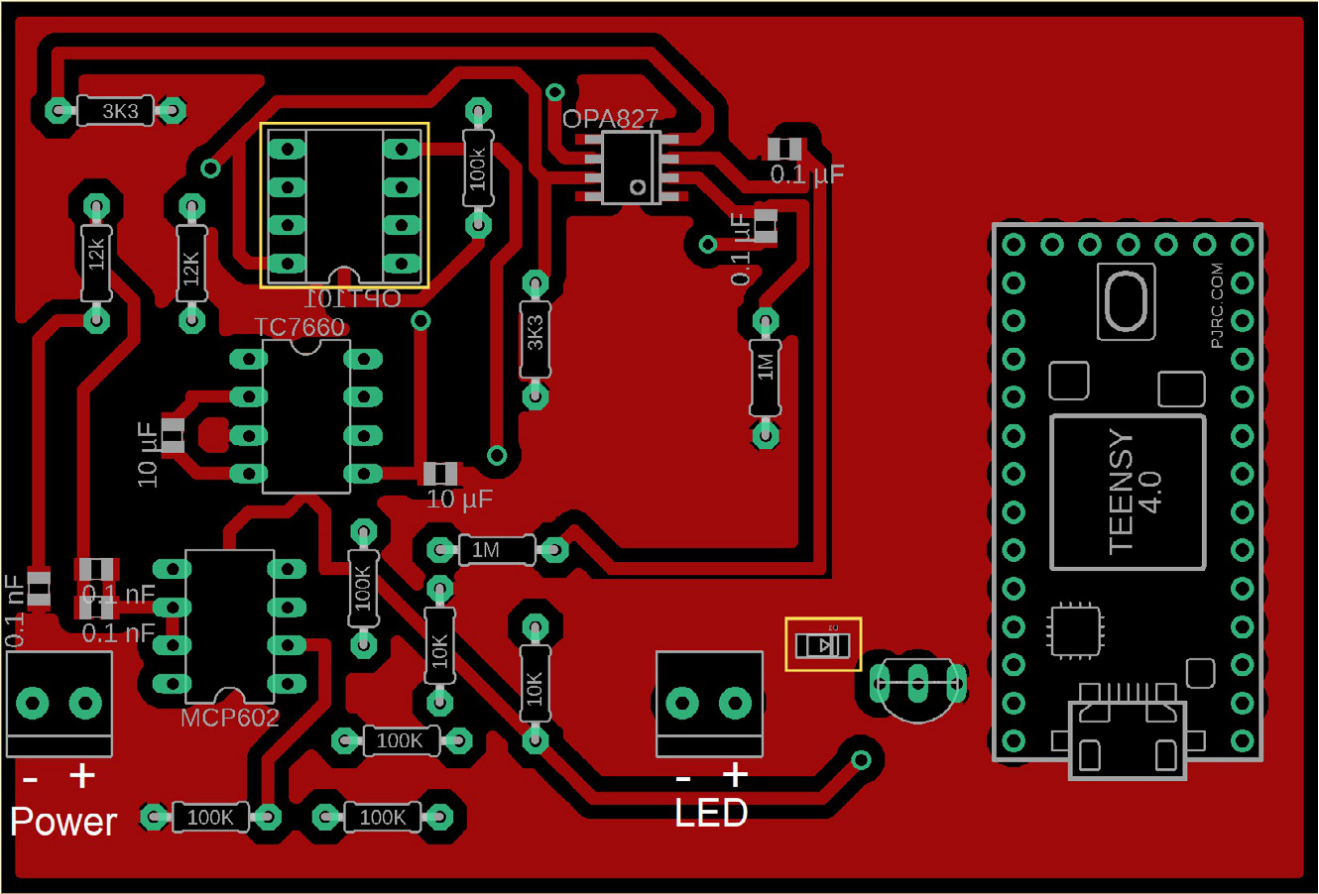
Diagram of the top side of the PCB, showing component orientations and values. The two components soldered to the underside of the PCB (DIP socket for OPT101 and 30 mA LED driver) are marked with yellow boxes. (For interpretation of the references to colour in this figure legend, the reader is referred to the web version of this article.)

The PCB is designed with wide traces to allow easy fabrication from double-sided copper PCB blanks using a milling machine. (The PCB used for the current work was fabricated from FR1 PCB blank using a Bantam Tools Desktop PCB milling machine with a 1/32″ endmill and 0.005″ PCB engraving bit; milling was carried out using the default feeds and speeds calculated by the accompanying control software). Alternatively PCB fabrication may be outsourced to a PCB service provider using the provided Gerber files. The locations, orientations and values of the components are shown in Fig. 5. A complete circuit diagram is provided in Fig. S2. All components other than the OPT101 amplified photodiode are soldered directly to the board; the OPT101 is mounted in an 8-pin DIP socket to simplify final assembly. The MCP602 and TC7660 may also be mounted in sockets, but this is not necessary. ~10 cm leads connect the LED to the terminal block on the PCB. Note, to protect the populated PCB against accidental liquid damage during use, it is advisable to spray it with a non-conductive, conformal, acrylic coating.

**Fig. 6:**
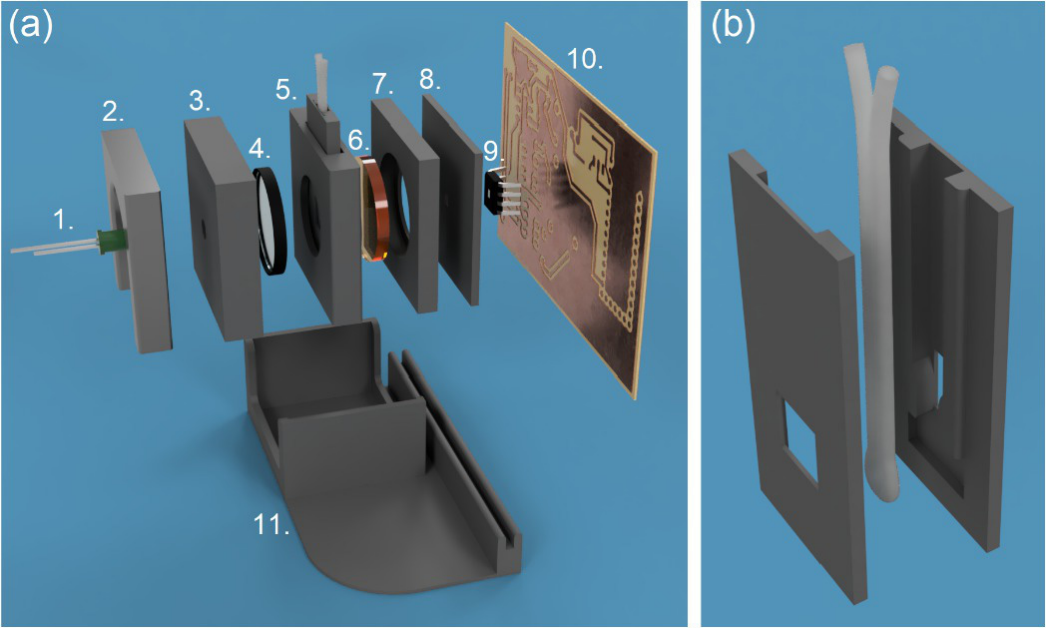
(a) Exploded-view of detector assembly, showing: LED (1), retaining clip (2), mounting plate for LED and short-pass filter (3), short-pass filter (4), flow-cell assembly (5), long-pass filter (6), mounting plate for long-pass filter (7), mounting plate for amplified photodiode (8), amplified photodiode (9) and PCB (10). (The PCB is shown unpopulated for clarity). The unit is assembled by stacking together parts 1 to 10 and slotting into the base-plate (11). The ring-mounted short-pass filter (4) presses into its mounting plate (3) with a friction fit, whereas the long-pass filter is held in place via two protrusions that extend from the flow-cell assembly. The LED and photodiode package are glued into their respective mounting plates. (b) Exploded view of flow cell. The flow-cell is constructed from a short (~10 cm), doubled-over length of 2-mm-OD, 1-mm-ID PTFE tubing glued between two 3D-printed retaining halves.

**Fig. 7:**
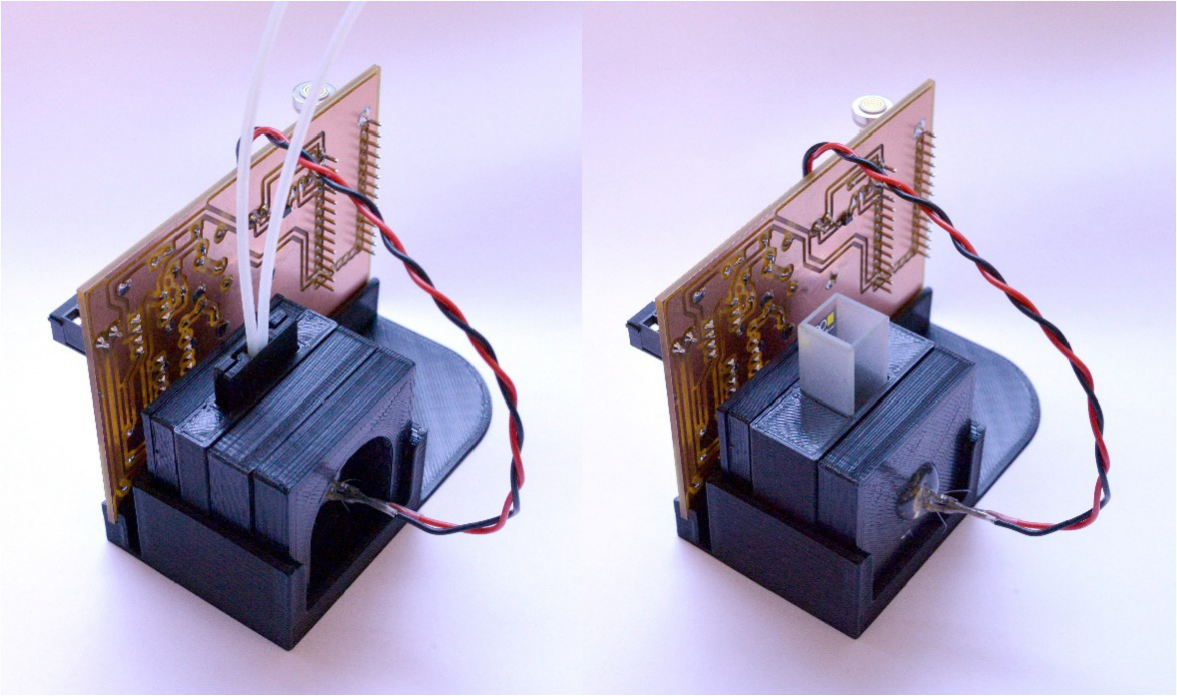
Detector assembly configured for use with a flow cell (left) and a cuvette (right), with the corresponding sample holders installed. The retaining clip (part 2 in Fig. 6a) is omitted in the cuvette configuration to compensate for the extra thickness of the cuvette holder.

All 3D-printed parts should be printed using black filament (polylactic acid [PLA] is suitable), using a layer thickness of 0.2 mm or less, and an infill of at least 10%. (The total printing time using an Anycubic i3 Mega 3D-printer was approximately four hours). Assembly of the instrument is straightforward, and is illustrated in Fig. 6a for the flow cell. The LED and OPT101 are glued into pre-formed recesses within their respective mounting plates using a small amount of hot glue. Next, the OPT101 is plugged into its socket on the PCB. The instrument is assembled by stacking the PCB and individual layers of the optical stack as shown in Fig. 6a, and slotting them – as a single unit – into the base; the optical stack is held together and retained in the base-plate by a friction fit. The flow-cell is formed using a ~10-cm length of 2-mm-OD, 1-mm-ID semi-transparent polytetrafluoroethylene (PTFE) tubing (228-4118, VWR) secured within the two 3D-printed halves using either hot glue or epoxy resin (see Fig. 6b). If required, the PTFE tubing could be replaced with more transparent fluorinated ethylene-propylene (FEP) tubing of the same dimensions, but this comes at a small cost premium. Fluidic connections to the internal tubing are made using nut-and-ferrule style Luer adaptors. The construction steps are almost identical for the cuvette holder, except the retaining clip is omitted to compensate for the greater depth of the cuvette holder. Side-by-side images of the two configurations are shown in Fig. 7.

### Operating instructions

First, connect the PCB to a 20–24 V unipolar power supply, using the terminal block labelled power in Fig. 5. Then select measurement settings within the firmware and flash the microcontroller. We recommend sticking to the default sample rate of 200 kHz and the default modulation frequency of ~410 Hz, but if necessary these can be changed by modifying the values of Fsig and Fsample in the pre-processor definitions of the file “FluorescenceSensor.ino”. The relevant locations are clearly commented.

Flashing of the firmware is most easily achieved using the Arduino IDE, which may be downloaded from https://www.arduino.cc/en/software. It is also necessary to download and install Teensyduino (https://www.pjrc.com/teensy/td_download.html) for compatibility with the Teensy 4.0. Flash the firmware by opening the firmware file “FluorescenceSensor.ino” within the Arduino IDE, connecting the device via a USB cable, and clicking the “Upload” button within the Arduino IDE. Once flashed, the microcontroller will output the measured lock-in amplitude $R_{2}$ at 0.1-s intervals, which can be read using the Arduino IDE’s serial monitor, or using the software front-end described below. Typically, it takes several minutes for the output of an LED to stabilise after initial switch-on [10]. For the LED used here, we (conservatively) recommend waiting at least fifteen minutes before beginning measurements; other LEDs may have shorter or longer stabilisation times.

**Fig. 8:**
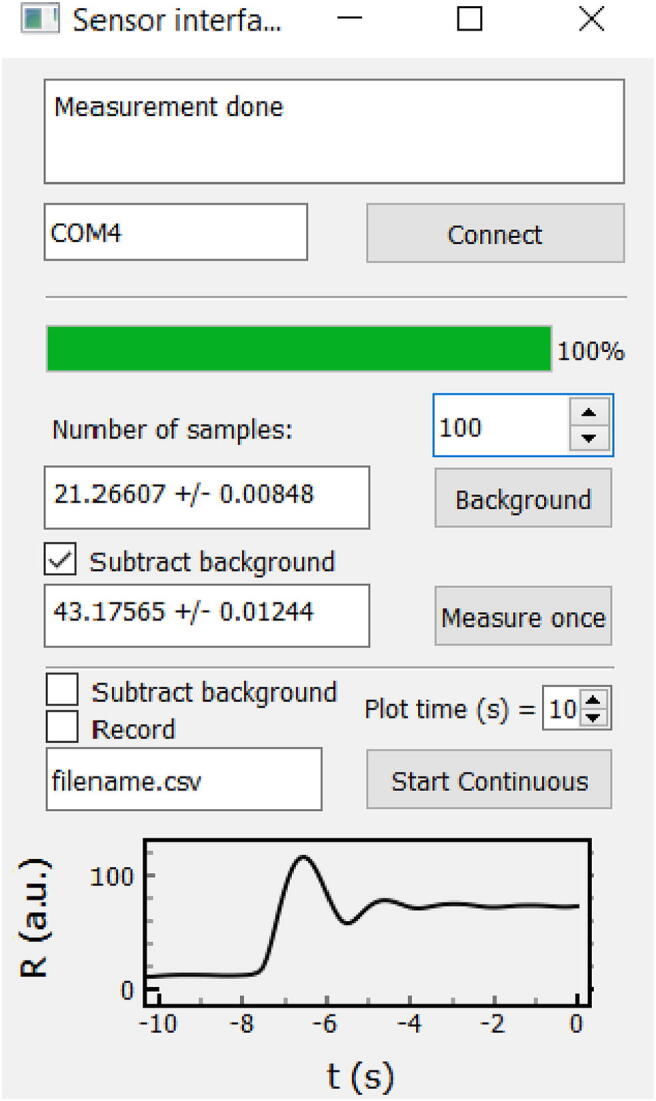
Screenshot of GUI software used for displaying measurements.

Measurements are most easily performed using the provided Python program. The software, “SensorInterface.py”, was written in Python 3.8.8, and requires installation of PyQt5, pyqtgraph, and numpy. A screenshot of the interface is shown in Fig. 8. After connecting the sensor to the PC via powered USB, and supplying 20–24 V DC power to the unit, connect to the device by typing in the correct serial address (COM4 in Fig. 8) and click “Connect”. The blank/background sample should then be loaded into the sample holder, and the desired number of 0.1-s readings to average over should be specified. To acquire a background measurement, click “Background”. The value of the background measurement and the standard deviation are displayed in the software’s graphical user interface (GUI). Load a test sample and click “Measure once” to acquire a single measurement; a checkbox determines whether or not background subtraction is carried out. Clicking “Start Continuous” causes the data stream to be continuously plotted within the GUI over a user-defined time range. Checking the “Record” checkbox causes the data stream to be saved to a comma separated variable (CSV) file; data is recorded both with and without a background subtraction. The continuous measurement can be interrupted at any time for a single measurement or a new background acquisition.

## Validation and characterisation

**Fig. 9:**
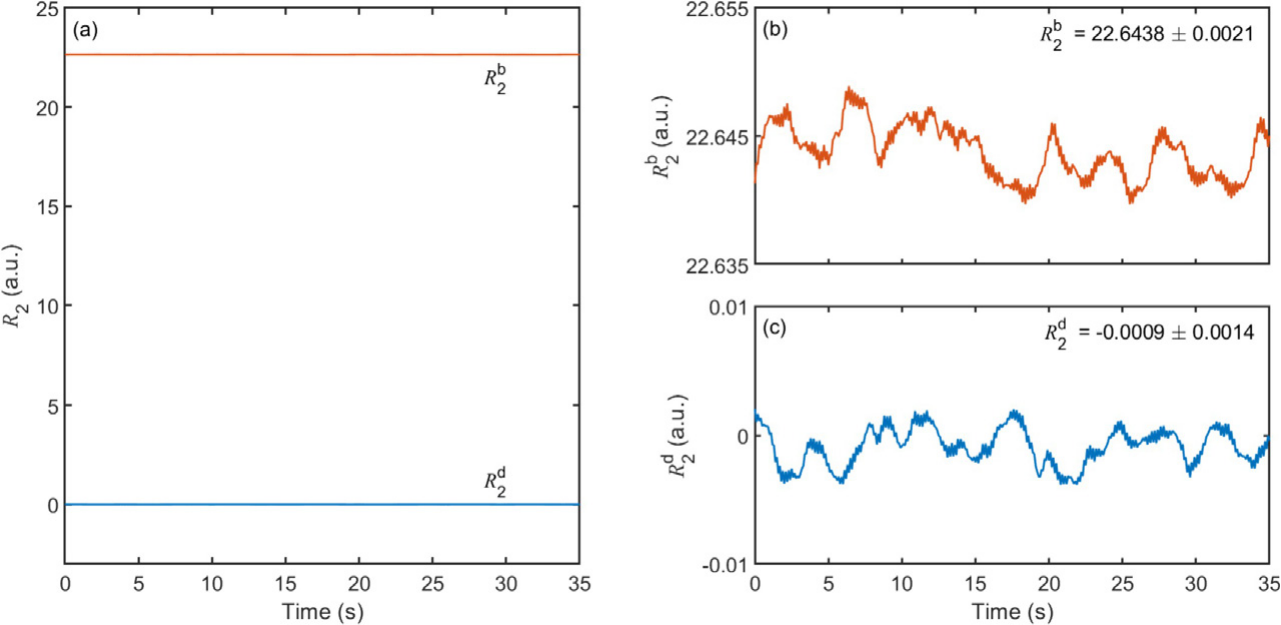
(a) Blank ($R_{2}^{b}$) and dark ($R_{2}^{d})$ signals recorded using the fluorescence detector with pure water in the flow-cell. (b), (c) zoomed-in traces of $R_{2}^{b}$ and $R_{2}^{d}$; inset numbers denote mean plus or minus one standard deviation.

Fig. 9 shows a “blank” signal $R_{2}^{b}$ versus t for a measurement obtained using pure water in the flow-cell, together with an equivalent “dark” signal $R_{2}^{d}$ obtained under nominally identical conditions but with the LED turned off. The two measurements were made under ambient conditions in a normally lit laboratory with no effort being made to shield the device from ambient light. The sample rate and square-wave modulation frequency were set to the default values of 200 kHz and ~410 Hz, respectively. $R_{2}^{d}$ and $R_{2}^{b}$ wandered around average values of −0.0009 and 22.6438 arbitrary units [a.u.] (see Fig. 9), respectively, with standard deviations $\sigma_{2}^{d}$ and $\sigma_{2}^{b}$ of 0.0014 a.u. and 0.0021 a.u. (Longer duration measurements of $R_{2}^{b}$ recorded without a cuvette in the sample holder revealed no evidence of systematic drift over periods of up to one hour, see e.g. Fig S4). The higher value of the blank signal is attributable to a combination of leakage of excitation light through the long-pass filter and filter auto-fluorescence, and could be substantially reduced by switching to an orthogonal detection geometry and/or using a better optimised combination of short- and long-pass filters to further attenuate the unwanted light, see Fig. S1b. $R_{2}^{b}$ represents a background signal that should be subtracted from any fluorescence measurement.

**Fig. 10:**
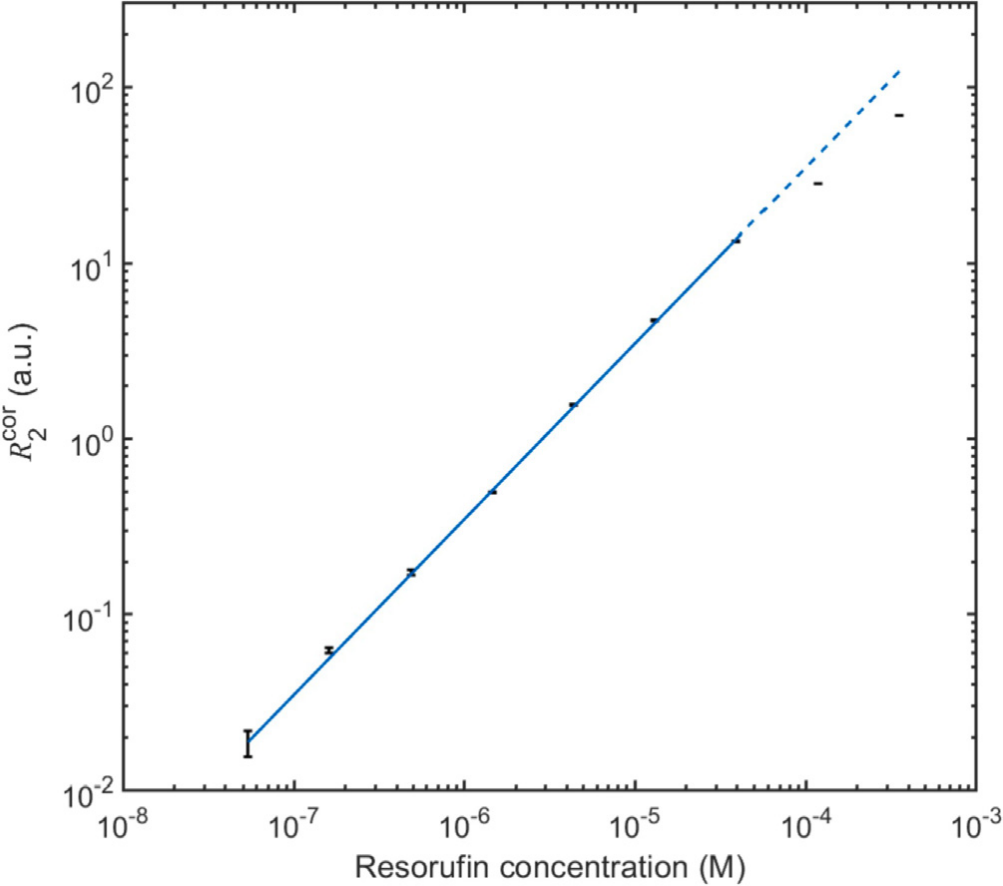
Background-corrected signal $R_{2}^{cor}=R_{2}-R_{2}^{b}$ versus resorufin concentration c over the range 54 nM to 0.35 mM, obtained using the flow-cell. The straight line represents a linear least-squares fit over the range 54 nM to 39 µM with an R^2^ value of 0.9990. Above 39 µM the relationship is sublinear due to the onset of dimerisation-induced self-quenching. The 2σ detection limit $R_{2}^{cor*}$ is 81 nM. Error bars span mean corrected signal plus or minus one standard deviation.

Fig. 10 shows the baseline-corrected signal $R_{2}^{cor}=R_{2}-R_{2}^{b}$ versus concentration for a serial dilution of the fluorescent molecule resorufin from 0.35 mM down to 54 nM. For each measurement, a solution of the dye molecule in pH-7.2 phosphate-buffered saline was injected into the flow-cell using a syringe, and the $R_{2}$-value was recorded for ten seconds (after stabilisation) to obtain the mean and standard deviation $\sigma_{2}$. The samples were measured in a randomised order, rinsing the flow cell with ultra-pure water and drying with compressed air between each measurement. $R_{2}^{cor}$ varied linearly with the concentration c of resorufin up to c=39μM, above which self-quenching led to a sub-linear response. $R_{2}$ could not be distinguished from the blank signal $R_{2}^{b}$ at the lowest tested resorufin concentration of 54 nM. Least-squares fitting of the data to a line of the form $R_{2}^{cor}=mc$ indicated a sensitivity m of 3.50 ± 0.05 × 10^5^ a.u./M, while extrapolation to $R_{2}^{cor}=2\sigma_{2}$ implied a 2σ detection limit $R_{2}^{cor*}$ of 65 nM.

**Fig. 11:**
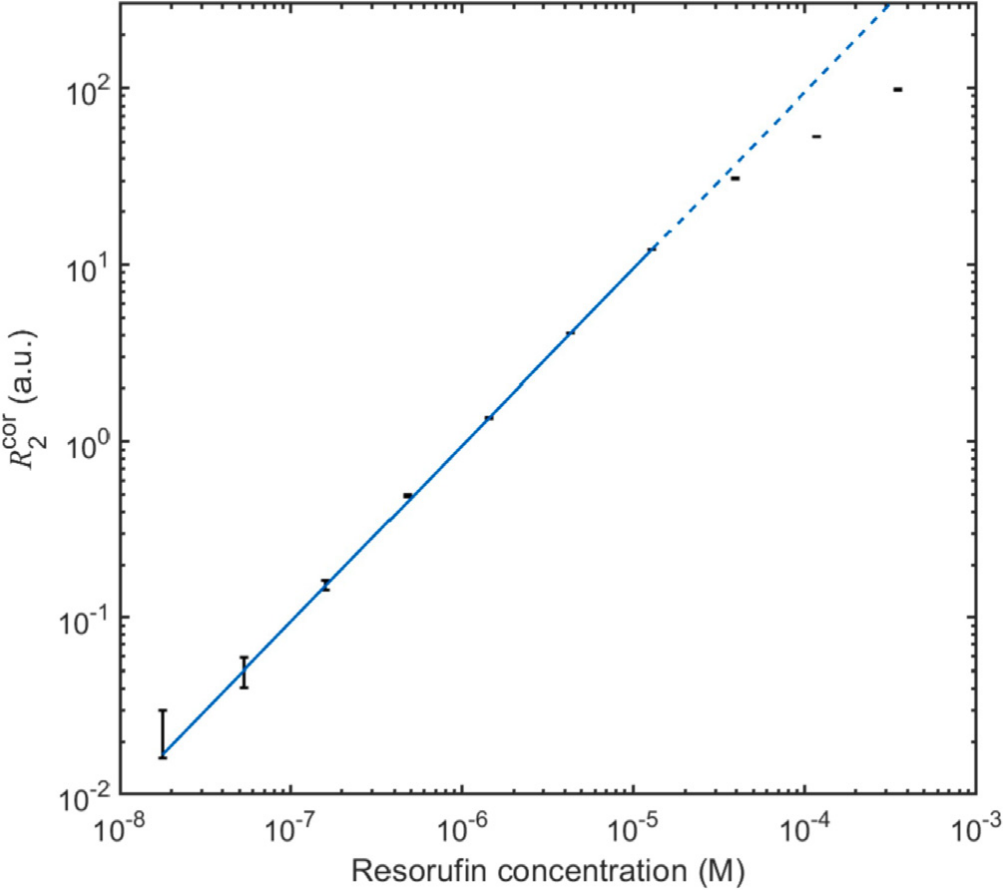
Background-corrected signal $R_{2}^{cor}=R_{2}-R_{2}^{b}$ versus resorufin concentration c over the range 18 nM to 0.35 mM, obtained using a 10-mm pathlength cuvette in the optical stack. The straight line represents a least-squares linear fit over the range 18 nM to 13 µM with an R^2^ value of 1.0000. Above 13 µM, a sublinear relationship is observed due to self-quenching and internal filtering of resorufin in the solution. The 2σ detection limit is 18 nM. Error bars span mean corrected signal plus or minus one standard deviation.

Fig. 11 shows equivalent data to Fig. 10, obtained using a 10-mm pathlength glass cuvette in place of the flow-cell. The extended pathlength provided by the cuvette resulted in a 2.7-fold increase in sensitivity to 9.47 ± 0.01 × 10^5^ a.u./M, with successful detection down to 18 nM and an estimated 2σ detection limit $R_{2}^{cor*}$ of 18 nM. Dividing the largest concentration in the linear range (0.35 mM) by the smallest detectable concentration (18 nM) indicated a dynamic range of approximately 2 × 10^4^, sufficient for most diagnostic applications.[11], [12], [13] (For comparative purposes, we show in Fig. S5 an equivalent plot obtained using the same OPT101 amplified photodiode and Teensy 4.0 microcontroller but without the signal conditioning circuit present or the lock-in algorithm active; the resulting dose–response data showed a much higher degree of scatter due to interference from uncontrolled ambient light, rendering the data unusable).

**Fig. 12:**
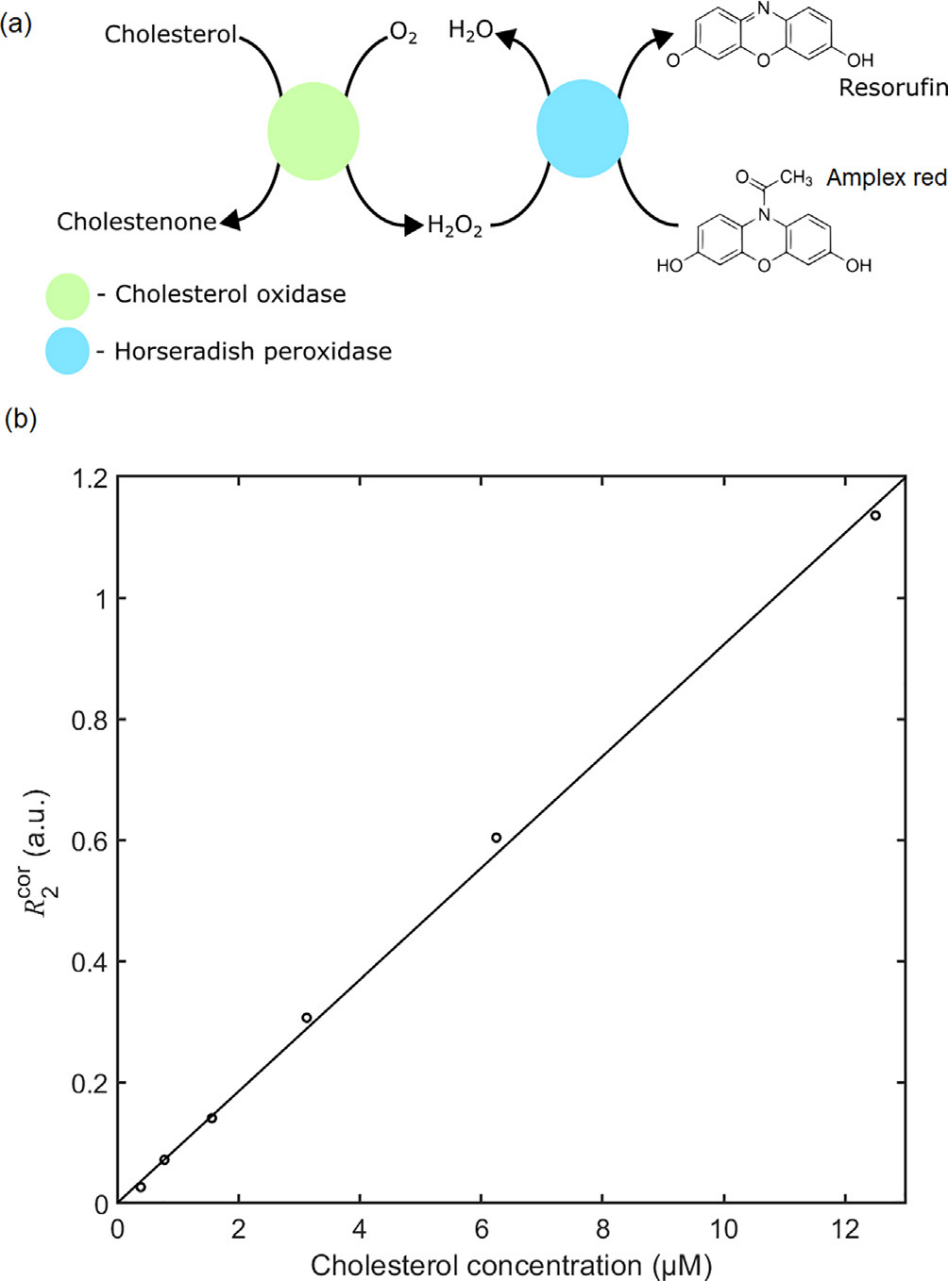
(a) Scheme showing chemical procedure used for cholesterol detection: cholesterol is oxidised by cholesterol oxidase to produce H_2_O_2_, which in turn is used by horseradish peroxidase (HRP) to convert Amplex Red into the fluorescent dye resorufin. (b) Corrected signal $R_{2}^{cor}=R_{2}-R_{2}^{b}$ vs cholesterol concentration c for the HRP assay. The straight line represents a least-squares optimised linear fit with R^2^ = 0.9984 and a 2σ detection limit of 200 nM.

The detector was further applied to the detection of cholesterol, using a fluorimetric assay based on the widely used probe Amplex Red (see Fig. 12a). The enzyme cholesterol oxidase catalyses the oxidation of cholesterol to cholestenone, with the concurrent reduction of oxygen to hydrogen peroxide (H_2_O_2_). In the presence of horseradish peroxidase, the generated H_2_O_2_ converts Amplex Red to the highly fluorescent dye molecule resorufin, with the measured fluorescence intensity being directly proportional to the initial cholesterol concentration. A stock solution of 4 U/ml cholesterol oxidase, 4 U/ml horseradish peroxidase (HRP), and 300 µM Amplex red was made up in PBS with 1% (v/v) TritonX-100. Six 1-ml aliquots were taken from the stock solution and combined with 1-ml cholesterol solutions with concentrations ranging from 780 nM to 25 µM. A negative control sample of 1% TritonX-100 in PBS (without cholesterol) was also prepared, and was used as the “blank” sample. The cholesterol solutions were prepared in PBS and contained 1% (v/v) TritonX-100. Hence seven 2-ml samples – each having a concentration of 2 U/ml HRP, 2 U/ml cholesterol oxidase and 150 µM Amplex Red – were obtained with cholesterol concentrations ranging from 0 to 12.5 µM cholesterol. The samples were left for 30 min at room temperature for the reaction to complete, and were then loaded sequentially into the flow-cell to determine the fluorescence intensity. For each sample the lock-in amplitude $R_{2}$ was recorded for 10 s after stabilisation.

Fig. 12b shows the baseline-corrected signal $R_{2}^{cor}=R_{2}-R_{2}^{b}$ versus cholesterol concentration c where $R_{2}^{b}$ is the background signal obtained with the negative control sample. Least-squares fitting of the data to a line of the form $R_{2}^{cor}=mc$ indicated a sensitivity m of 9.2 × 10^4^ a.u./M with an R^2^ linear correlation coefficient of 0.9984, while extrapolation to $R_{2}^{cor}=2\sigma_{2}^{b}$ implied a detection limit $R_{2}^{cor*}$ of 200 nM – some 25,000 times lower than the typical 5-mM concentration of cholesterol in human serum (and hence sufficient for diagnostic applications) [11], [12].

## Human and animal rights

No human or animal subjects or samples were used.

## Declaration of Competing Interest

The authors declare that they have no known competing financial interests or personal relationships that could have appeared to influence the work reported in this paper.
