# Supplementary material for: A sensitive and compact optical detector based on digital lock-in amplification
Source: HardwareX. 2021 Sep 2;10:e00228. doi: 10.1016/j.ohx.2021.e00228 (PMC9123480; doi:10.1016/j.ohx.2021.e00228)
Supplement: Supplementary data 1 [file mmc1.pdf]

SUPPORTING INFORMATION

A sensitive and compact optical detector based on digital lock-in amplification

Andrew J. Harvie*, Surendra K. Yadav, John C. de Mello*

Department of Chemistry, NTNU, Trondheim, Norway

email: andrew.j.harvie@ntnu.no and john.demello@ntnu.no

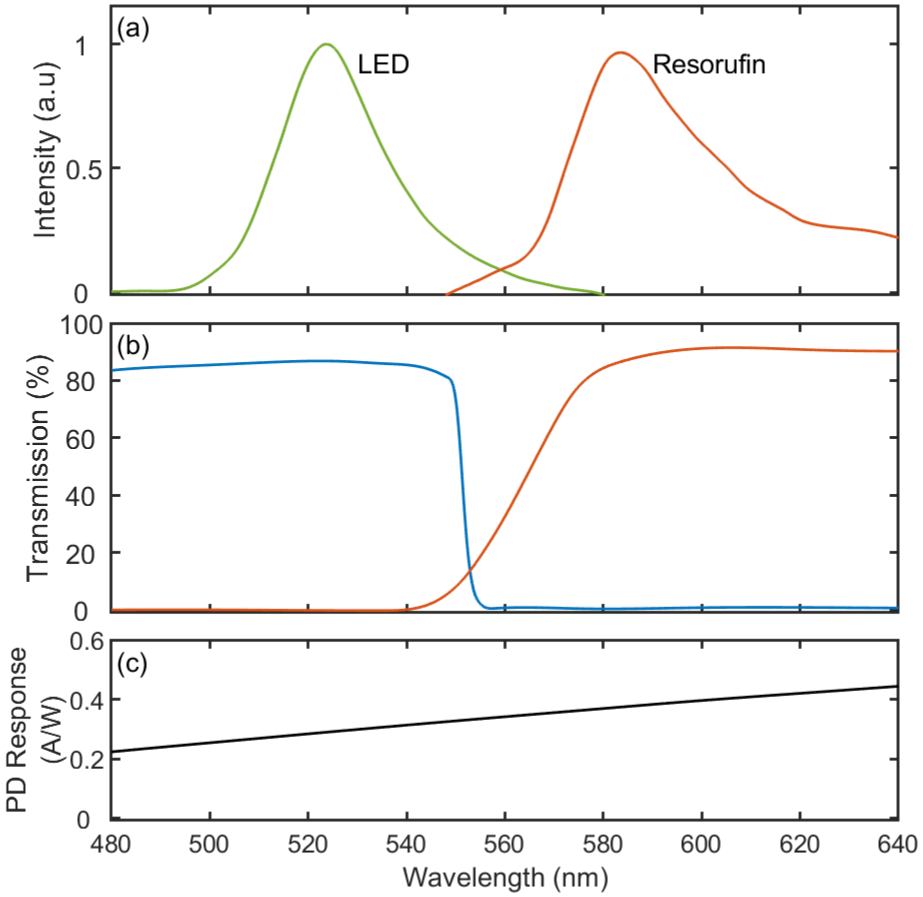

Fig. S1 Spectral characteristics of optical and optoelectronic components. (a) Normalised emission spectra of LED525L light-emitting diode (green) and of resorufin (red). (b) Transmission spectra of FES0550 short-pass filter (blue) and FGL570 long-pass filter (red). The small overlap in the pass bands gives rise to a background signal R_2^{b} that must be subtracted from each fluorescence measurement. The background signal could be reduced by choosing a pair of filters with a smaller overlap at the expense of a small reduction in fluorescence signal. (c) Responsivity of OPT101 photodiode (PD). Spectra taken from component datasheets.

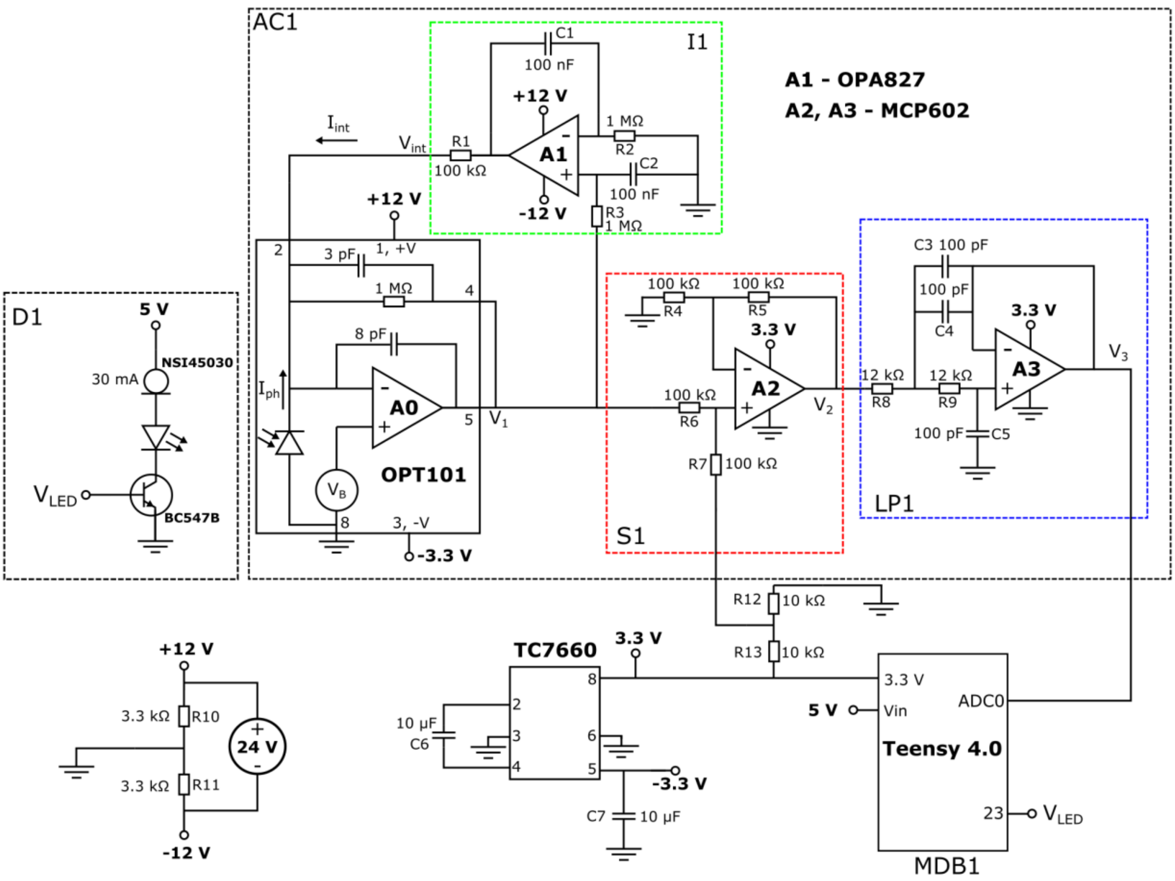

Fig. S2 Complete circuit diagram for optical detector. The circuit comprises an LED driver (D1), analogue circuitry (AC1) for signal acquisition and conditioning, and a Teesny 4.0 microcontroller development board (MDB1). AC1 comprises an OPT101 amplified photodiode with a non-inverting integrator (I1) included in its feedback path for DC compensation, a summing amplifier S1 that adds a +1.6 V DC offset to the (DC-compensated) input signal, and a 94 kHz low-pass filter (LP1) that suppresses noise and high-frequency components above the 100-kHz Nyquist frequency of the signal. MDB1 is used for analogue-to-digital conversion, digital signal processing, and to generate a 3.3 V square-wave signal V_{LED} that controls the LED driver via the base terminal of the BC547B transistor.

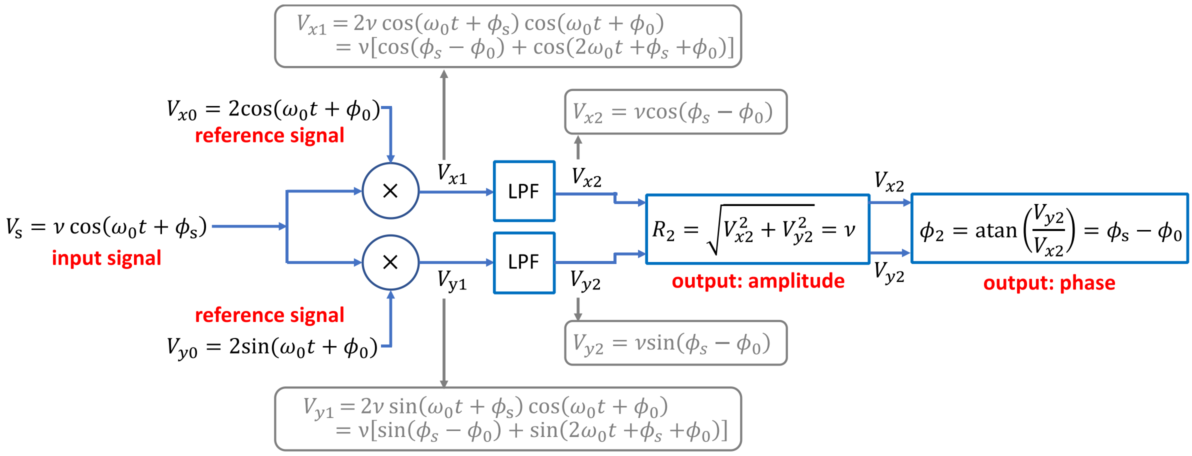

Fig. S3 Block diagram illustrating the lock-in procedure for an incoming signal of equal frequency to the reference signals. An input signal V_S of frequency ω_0, phase ϕ_s and amplitude v is passed into two separate channels. In the first channel V_S is multiplied by a reference signal V_{x0} of the form $V_{x0} = 2cos(\omega_0 t + \phi_0)$ and passed through a low-pass filter (LPF), yielding an output $V_{x2} = vcos(\phi_S - \phi_0)$; in the second channel V_S is multiplied by a reference signal of the form $V_{y0} = 2sin\,(\omega_0 t + \phi_0)$ and passed through a second low-pass filter, yielding an output $V_{y2} = vsin(\phi_S - \phi_0)$. V_{x2} and V_{y2} are vectorially combined to yield a final output $R_2 = \sqrt{V_{x2}V_{x2} + V_{y2}V_{y2}}$ that is equal to the amplitude v of the input signal V_S. For an arbitrary periodic input signal, R_2 is equal to the amplitude of the first harmonic of the input signal (and hence is proportional to the amplitude of the input signal). If required, the phase of the input signal relative to the reference signals may also be calculated from $\phi_2 = \text{atan}\,(V_{y2}/V_{x2})$. Contributions from input signals at frequencies away from ω_0 are heavily attenuated by the low pass filter and do not significantly affect R_2 or ϕ_2.

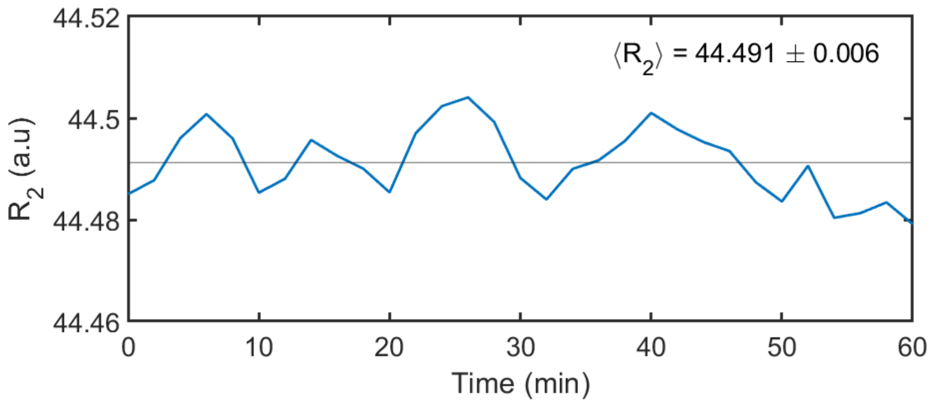

Fig. S4 Lock-in signal versus time for an illustrative blank measurement. The output was recorded at two-minute intervals over the course of one hour, with the cuvette holder installed but the cuvette missing. The signal wandered about a mean value of 44.491 a.u. with a standard deviation of 0.006 a.u., corresponding to a relative standard deviation of 0.1%. There is no sign of systematic drift.

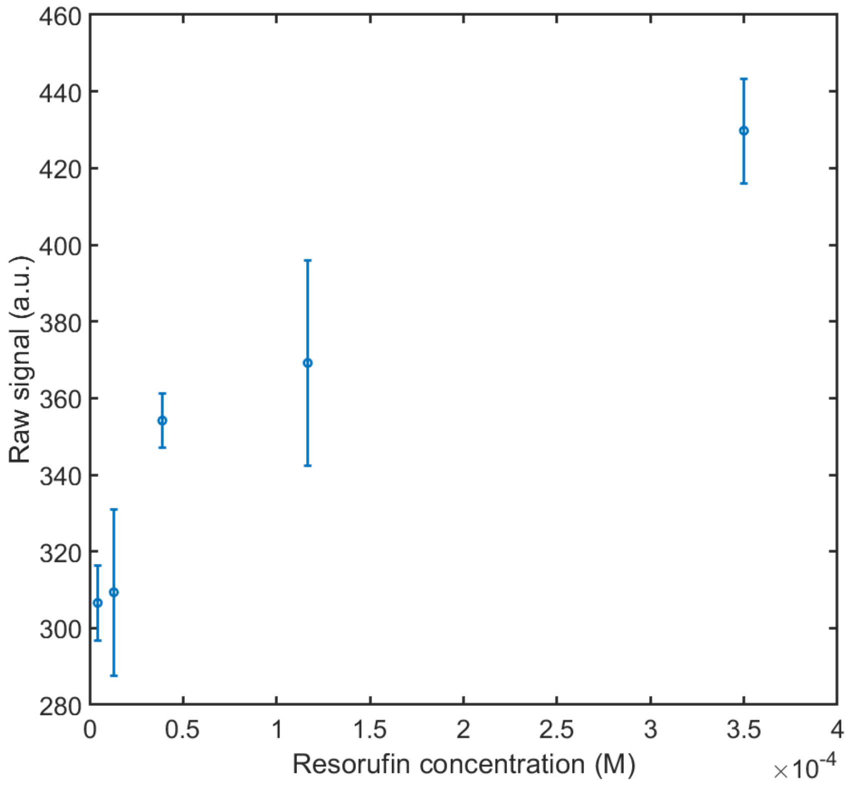

Fig. S5 DC signal recorded by the ADC versus resorufin concentration. The data was obtained by connecting the OPT101 amplified photodiode directly to an analogue input pin of the microcontroller, operating the LED at a fixed current of 30 mA. The ADC was operated at 200 kHz, and for each concentration the signal was averaged over a period of 10 s. The filled circles denote the mean signal, while the error bars span one standard deviation. Large fluctuations in the measured signals were observed due to interference from background light, with the data showing an average relative standard deviation (RSD) of ~4 % compared to around ~0.01 % when the signal conditioning circuit and lock-in algorithm were used, see Fig. 9 and Fig. S4.

Appendix S1 - Brief explanation of analogue conditioning circuit

The first stage of the conditioning circuit (see Fig. S2) is a feedback-based compensation stage that removes any DC offset in the output voltage V_1 of the OPT101 due to ambient light or non-ideality of A0 [S1]. The inclusion of the compensation stage allows the OPT101 to operate under conditions of high ambient illumination without saturating A0, A2, A3 or ADC0. DC compensation is achieved by passing V_1 into an op-amp based non-inverting integrator I1 that integrates signals close to DC, while attenuating high frequency signals. For a typical scenario in which the photodiode is incompletely shielded from stray light, the photocurrent I_{ph} exhibits a negative DC offset ΔI due to ambient illumination, and (since the OPT101 is configured as an inverting amplifier) V_1 therefore initially exhibits a positive DC offset ΔV. This positive offset may be very much larger than the amplitude of the target signal and, unless corrected for, would make signal recovery difficult. Owing to the positive sign of ΔV, the integrator generates a steadily increasing positive output voltage V_{int} that in turn drives a steadily increasing positive correction current $I_{\mathrm{int}} = V_{\mathrm{int}}/R_1$ into the summing junction of A0 (pin 2 of the OPT101). I_{int} partially cancels ΔI and so causes ΔV to decrease, while leaving the high frequency components of V_1 unchanged. V_{int} and I_{int} continue to rise in magnitude until the DC component of I_{ph} is fully compensated and ΔV is therefore zero.

The op-amp A1 in integrator I1 can output a maximum voltage of 12 V; as R1 has a value of 100 kΩ, the compensation circuit can supply up to 120 μA of correction current, which is approximately 40 times larger than the photocurrent (3.3 μA) needed to saturate ADC0. The compensation circuit therefore allows the lock-in amplifier to operate under high DC light levels that would otherwise saturate ADC0 and prevent signal recovery. The circuit can only be used with modulation frequencies that are substantially higher than the integrator’s 16-Hz cut-off frequency (since lower frequencies are fed-back to the summing terminal of A0). However, this is not a significant limitation since high modulation frequencies are usually preferred to reduce “flicker noise” [S2] and improve sensitivity. For the work reported here, we use a modulation frequency of $\sim$410 Hz.

Following DC compensation V_1 is a bipolar signal with a mean value of zero, whereas ADC0 on the Teensy microcontroller can only accept positive input voltages in the range 0 to 3.3 V. Hence to bring the signal into a range that can be measured by ADC0, we add a fixed offset of 1.6 V to V_1 by means of another op-amp A2, which is configured as a unity-gain non-inverting summing amplifier (S1). The resulting output signal V_2 is passed to a third op-amp A3, which is configured as a unity gain low-pass filter (LP1) with a cut-off frequency of 94 kHz, i.e. slightly less than half the 200-kHz sampling frequency used by ADC0. The low-pass filter suppresses noise and interferences above the 100-kHz Nyquist frequency and therefore acts as an anti-aliasing filter that prevents sampling artefacts.

Appendix S2 - Brief overview of lock-in detection

When a signal is masked by noise and other interferences, it is helpful to modulate the light-source at a fixed angular frequency ω_{mod} so as to impose a matching frequency on the fluorescence signal. Band-pass filtering of the electrical signal generated by the photodiode can then be used to reject interferences at frequencies away from ω_{mod}, enabling recovery of the target signal. The narrower the bandwidth of the filter – i.e. the smaller the range of frequencies the filter passes – the more completely interferences away from ω_{mod} are rejected, and hence the more easily the target signal can be detected.

A band-pass filter may be characterised in terms of its Quality factor $Q = \omega_{\mathrm{c}}/\Delta\omega$ where ω_{c} is its centre frequency and $\Delta\omega$ is its bandwidth, with higher values of Q implying better rejection of unwanted signals at frequencies close to (but not equal to) ω_{c}. High Q filters typically exhibit poor stability, [S3] and in practice it is difficult to design band-pass filters with Q values much greater than 100, which limits how effectively noise and other interferences can be rejected by band-pass filtering. However, a related device known as a lock-in amplifier (LIA) [S4] can deliver effective Q values that are much higher than this (up to a million or more), resulting in better rejection of noise and interferences.

An LIA tuned to an angular frequency ω_0 transforms an AC input signal of frequency ω_0 into a *DC* output signal that is proportional in size to the amplitude of the input signal. Note, this behaviour differs from a band-pass filter, which converts an AC input signal at its centre frequency into an *AC* output signal of matching frequency (but possibly different amplitude and phase depending on the specific design) [S5].

The lock-in amplifier generates two internal reference signals V_{x0} and V_{y0} of frequency ω_0, which are separated in phase by $\pi/2$ radians:

$$V_{x0} = 2\cos(\omega_0 t + \phi_0) \qquad \text{(S1a)}$$

$$V_{y0} = 2\cos(\omega_0 t + \phi_0 - \pi/2) = 2\sin(\omega_0 t + \phi_0) \qquad \text{(S1b)}$$

where ϕ_0 is a constant phase offset and the pre-factor of two is chosen for algebraic convenience.

For simplicity, we begin by assuming the incoming signal V_{s} is a pure cosinusoid of frequency ω_{s}, phase ϕ_{s} and amplitude v:

$$V_{\mathrm{s}} = v\cos(\omega_{\mathrm{s}} t + \phi_{\mathrm{s}}). \qquad \text{(S2)}$$

In the first stage of lock-in amplification, the input signal V_{s} is multiplied by the two reference signals, generating two intermediate signals, V_{x1} and V_{y1}. In the case of the V_{x0} reference signal, for instance, we obtain an intermediate output voltage

$$V_{x1} = 2v\cos(\omega_{\mathrm{s}} t + \phi_{\mathrm{s}})\cos(\omega_0 t + \phi_0), \qquad \text{(S3)}$$

which may be rewritten in the form

$$V_{x1} = \mathrm{v}[\cos((\omega_{\mathrm{s}} - \omega_0)\, t + (\phi_{\mathrm{s}} - \phi_0)) + \cos((\omega_{\mathrm{s}} + \omega_0)\, t + (\phi_{\mathrm{s}} + \phi_0))]. \qquad \text{(S4)}$$

For the typical case $\omega_{\mathrm{s}} \neq \omega_0$, we obtain two cosinusoidal components at the sum and difference frequencies of ω_{s} and ω_0. In the second stage of lock-in amplification we pass the intermediate output signal V_{x1} through a low-pass filter with a cut-off frequency ω^* that is much smaller than $|\omega_s - \omega_0|$.

The filter heavily attenuates both frequency components of V_{x1}, generating an output signal V_{x2} that is close to zero.

Only when the frequency of the incoming signal is closely matched to the frequency of the reference signal ($|\omega_s - \omega_0| \ll \omega^*$) do we obtain a non-zero output. For the special case $\omega_s = \omega_0$ (see Fig. S3), the output of the multiplier is given by:

$$V_{x1} = \mathrm{v}[\cos(\phi_s - \phi_0) + \cos(2\omega_0 t + \phi_s + \phi_0)], \tag{S5}$$

where the first term in the square bracket is a constant. After DC filtering, we therefore obtain

$$V_{x2} = \mathrm{v}\cos(\phi_s - \phi_0), \tag{S6}$$

which is proportional to both the amplitude v of the incoming signal and the cosine of the phase difference between V_s and V_{x0}.

Carrying out an equivalent analysis with the second reference signal V_{y0}, we obtain

$$V_{y2} = \mathrm{v}\sin(\phi_s - \phi_0). \tag{S7}$$

By solving Eqs. (S6) and (S7) simultaneously we obtain expressions for the amplitude and phase of the input signal V_s in terms of V_{x2} and V_{y2}:

$$R_2 = \sqrt{V_{x2}^2 + V_{y2}^2} = \nu \tag{S8}$$

and

$$\phi_2 = \mathrm{atan}\left(\frac{V_{y2}}{V_{x2}}\right) = \phi_s - \phi_0 \tag{S9}$$

From the above analysis it is evident that a lock-in amplifier set to a reference frequency ω_0 is 'blind' to all signals that differ from ω_0 by substantially more than the low-pass filter cut-off frequency ω^*. Hence, if ω_0 is set to the fundamental frequency ω_s of an incoming periodic signal (which need not be sinusoidal), the output R_2 from the lock-in amplifier will equal the amplitude of the first harmonic component of that signal. Signals at frequencies away from ω_s – whether due to higher harmonics of the incoming signal, noise or other interferences – are heavily attenuated by the long-pass filter and do not significantly affect the value of R_2.

The effective bandwidth of a lock-in amplifier is equal to twice the cut-off frequency of the low-pass filter (since signal frequencies just above and just below ω_0 are transformed to near-DC intermediate signals that partially survive the filtering step). For our specific LIA implementation the cut-off frequency is fixed at 0.25 Hz, while the modulation frequency is fixed at ~410 Hz. The effective bandwidth of the LIA is therefore 0.5 Hz (= 2 × 0.25 Hz) compared to ~4.1 Hz (= 410 Hz/100) for a very narrow $Q \sim 100$ band-pass filter such as Linear Technology's 'ultraselective' 8th-order elliptic bandpass filter LTC1164-8. [S6] The LIA as configured here therefore provides substantially better noise rejection than high-end band-pass filters. Moreover, if required, the LIA's noise rejection may be further improved without the need for any hardware changes by simply reducing the cut-off frequency of the long-pass filter (at the expense of a longer settling time).

Finally, we note that in the above analysis we assumed the reference signals and the incoming signal had constant (time-invariant) phases, implying a fixed phase difference between them. In practice many real signals are susceptible to jitter and drift which, left uncorrected, introduce noise and error into the lock-in measurement. To avoid these problems, the reference signals and incoming signal

should be synchronised so that jitter or drift in the incoming signal is perfectly mirrored in the reference signals, resulting in a constant phase difference between them. This may be achieved by means of a phase-locked-loop [S7] that causes the reference signals to ‘follow’ the incoming signal or – more simply – by using one of the two internally generated reference signals to modulate the incoming signal. The latter approach is used in our LIA implementation, with the microcontroller generating a square-wave output voltage that is phase-locked to the x-channel reference signal (Eq. S1a). The square-wave voltage toggles the light-source on and off, and so modulates the fluorescence signal at the reference frequency ω_0.

References

[S1] M. Stitt, W. Meinel, OPT201 Photodiode amplifier rejects ambient light, Burr-Brown Application Note, 1993.

[S2] E. Milotti, 1/f noise: a pedagogical review, 2002. https://doi.org/10.1021/acsnano.5b06163.

[S3] Maxim Integrated, A Beginner's Guide to Filter Topologies, 2003.

[S4] K. Kishore, S.A. Akbar, Evolution of Lock-In Amplifier as Portable Sensor Interface Platform: A Review, IEEE Sens. J. 20 (2020) 10345–10354. https://doi.org/10.1109/JSEN.2020.2993309.

[S5] R. Mancini, Op-Amps for Everyone, 2nd ed., Newnes, 2003.

[S6] Linear Technology, LTC1164-8 Datasheet, (1995).

[S7] I. Collins, Phase-Locked Loop (PLL) Fundamentals, Analog Dialogue. (2018) 1–6.
